# Health Tract, No. 252

**Published:** 1866-06

**Authors:** 


HEALTH TRACT, No. 252.

### NIGHT WORK.

Many of the most brutal murders, and greatest crimes perpetrated in the city of New York, are committed by persons under twenty-five years of age; this shows a very early corruption of morals and as an eminent jurist once said, is easily traceable to the habit of being from home after dark. Lord Shaftsbury statod fron the bench, that in nearly all the cases of great crimes which came before him the evidence showed that the moral character became vitiated between the ages of eight and sixteen; these two terrible facts put together should make every city parent especially, tremble; and if it should lead to the adoption of the following suggestions, it will save many a heart from going down in sorrow to the grave, from an embittered old age.

Do not allow your children to form the habit of "going home," to spend the night with their companions, no not once in a year.

Keep them out of the street after sun down, unless you are with them.

Do all that is possible to have a loving, cheerful and happy fireside, as a means of winning them from the street. Much can be done in this direction by providing amusements, and having the children occupied in something which is interesting, profitable or new.

Keep the birthdays; let them be occasions of harmless festivities: arrange that all the holidays too,shall be observed appropriately. Little parties given now and then to those of their own age, is a source of much delight to children, and they may be so conducted as to be of great benefit morally, socially and physically.

Let the father and mother remember that the exhibition before their children of a loving, affectionate and quiet deportment towards one another in the home circle, is a powerful bond of union in a family; the very sight of it wakes up affectionate sympathies in the hearts of children, and cherishes the same delightful feelings in themselves; and soon the house becomes a home of love and quiet delight; within half a mile of us, there are quite a number of families of this sort, some of them among the wealthiest in the city, but it is singular to observe that in almost every case it is in consequence of the mother's all pervading influence, mothers who are quiet, gentle, lady like, but firm in the right always. Many homes are made distasteful to children by incessant restrictions and criticisms; by innumerable rules and regulations. A household is better regulated by an affectionate pliancy, than by an inflexible rigidity; yielding in non-essentials, but firm as a rock in all questions of right and wrong. The night work from eight to sixteen determines the life character of millions.

